# Connexin43 and Its Regulation of Astrocyte Gap Junction Function: Influencing Depression Progression by Mediating Electrical and Chemical Signals

**DOI:** 10.1111/cns.70600

**Published:** 2025-09-05

**Authors:** Hongbin Wang, Cong Chen, Yuting Lin, Zhifeng Tian, Zihan Yan, Xuan Zeng, Yantao Yang, Meiyu Lin, Qidi Ai, Xuan Liu, Songwei Yang, Naihong Chen

**Affiliations:** ^1^ Hunan Engineering Technology Center of Standardization and Function of Chinese Herbal Decoction Pieces, School of Pharmacy Hunan University of Chinese Medicine Changsha China; ^2^ School of Pharmacy Hunan University of Chinese Medicine Changsha China; ^3^ School of Traditional Chinese Medicine Hunan University of Chinese Medicine Changsha China; ^4^ Institute of Innovation and Applied Research in Chinese Medicine Hunan University of Chinese Medicine Changsha China; ^5^ Institute of Materia Medica Chinese Academy of Medical Sciences & Peking Union Medical College Beijing China

**Keywords:** astrocyte, chemical signal, connexin43, depression, electrical signal, gap junction

## Abstract

**Background:**

Depression is a common mental illness with a high relapse rate, which has a serious negative impact on national economic development and happiness. At present, the pathogenesis of depression is still unclear, and there are inevitable limitations in first‐line clinical treatment. Therefore, it is very important to clarify the pathological mechanism of depression for the development of safe and effective antidepressants.

**Objective:**

In recent years, considerable research has shown that connexin43 (Cx43) and its regulated astrocyte gap junction (GJ) dysfunction are closely related to the occurrence and development of depression. This review aims to summarize the mechanisms by which Cx43 and its‐mediated astrocytic GJs contribute to depression progression, focusing on their regulatory roles in transmitting electrical signals (K^+^, Ca^2+^) and chemical signals (neurotransmitters, inflammatory factors). This work provides theoretical foundations for elucidating the pathological mechanisms of depression and developing novel antidepressant therapies.

**Conclusion:**

Cx43 and the gap junctions it regulates in astrocytes play a pivotal role in the pathophysiology of depression by influencing both electrical and chemical signaling between neurons. Further investigation into its mechanisms may offer novel therapeutic approaches for depression.

## Graphical abstract

A close relationship exists between Cx43‐mediated gap junction channels in astrocytes and depression. The astrocytic network formed through gap junction channels, in collaboration with neurons, participates in mediating intercellular electrochemical signaling within the nervous system. It plays a crucial role in maintaining the nervous system's environmental homeostasis, regulating synaptic function, and sustaining the establishment of brain function.

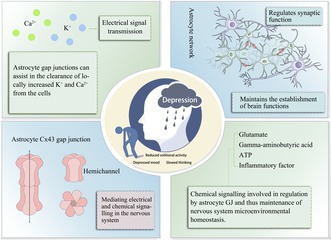

## 1 Introduction

Depression is an increasingly serious mental illness worldwide. Its prevalence and harm have attracted widespread attention from the international community. According to multiple data from the World Health Organization (WHO) and other research institutions, the prevalence and impact of depression worldwide have shown a significant increasing trend. About 340 million to 350 million people suffer from depression worldwide, accounting for about 4% of the global population [1]. Depression is also one of the leading causes of disability worldwide, accounting for 7.5% of all disease burdens [2]. People with depression are at higher risk of suicide, with approximately 800,000 deaths annually due to depression [3]. However, most patients currently do not receive timely diagnosis and treatment, and first‐line antidepressant treatment is ineffective for most patients. Therefore, the prevention, diagnosis, and treatment of depression need to be improved urgently. Further understanding of the pathogenesis of depression is of great significance for exploring new therapeutic targets and developing new antidepressants.

Astrocytes are one of the most abundant and functionally complex glial cell types in the central nervous system (CNS). Astrocytes not only provide support and protection for neurons, but also participate in a variety of key physiological processes, including maintaining brain homeostasis, regulating signal transmission, participating in the formation of the blood–brain barrier, and repairing nerve damage and disease [4]. Connexin43 (Cx43) is the most abundant connexin protein expressed in astrocytes, which plays a key role in the formation of gap junctions (GJ) and hemi‐channels (HC) [5]. Cx43 is able to achieve rapid synchronization and transmission of electrical signals between cells through its mediated GJ and HC, promoting intercellular communication and signal diffusion [6]. Astrocytes successfully syncytial through GJ junctions formed by Cx43, which allows direct exchange of ions, metabolites, and second messengers between cells, thus regulating neuronal activity [7]. In addition, astrocytes can release glial transmitters through GJ‐mediated communication, which can regulate synaptic chemical signal transduction, thus maintaining the functional homeostasis of the nervous system and preventing the occurrence and development of depression [8]. Neurons and astrocytes can also form a dynamic interaction network through GJ, regulate synaptic function, and maintain brain function establishment. In conclusion, Cx43 and astrocyte GJ are involved in regulating the transmission of electrical and chemical signals in the nervous system and play a key role in neural signal transmission and brain function.

At present, there are many hypotheses about the pathogenesis of depression, including the monoamine hypothesis, brain‐derived neurotrophic factor (BDNF) hypothesis, hypothalamic–pituitary–adrenal (HPA) axis hypothesis, and neuroinflammation hypothesis. Recent studies have demonstrated that astrocyte Cx43 dysfunction plays an important role in the pathogenesis of depression [9]. GJ dysfunction in astrocytes is closely associated with decreased Cx43 expression in patients with depression. It was found that Cx43 expression in the prefrontal cortex was significantly reduced in patients with major depressive disorder (MDD), leading to GJ dysfunction [10]. Dysfunction of Cx43 can prevent astrocytes from normally participating in neuron‐astrocyte signal transmission, triggering a series of pathological changes. For example, GJ dysfunction can lead to abnormal neurotransmitter metabolism [11], exacerbated neuroinflammation [12], and abnormal electrical signal transmission [13]. To sum up, this paper aims to investigate the impact of Cx43 and its regulated astrocytes GJ on electrical and chemical signal transmission in the nervous system and further analyze its impact on the process of depression, providing new ideas for further clarifying the pathogenesis of depression.

## 2 Cx43 and Astrocyte GJ

### 2.1 Structure and Functions of Cx43

**FIGURE 1:**
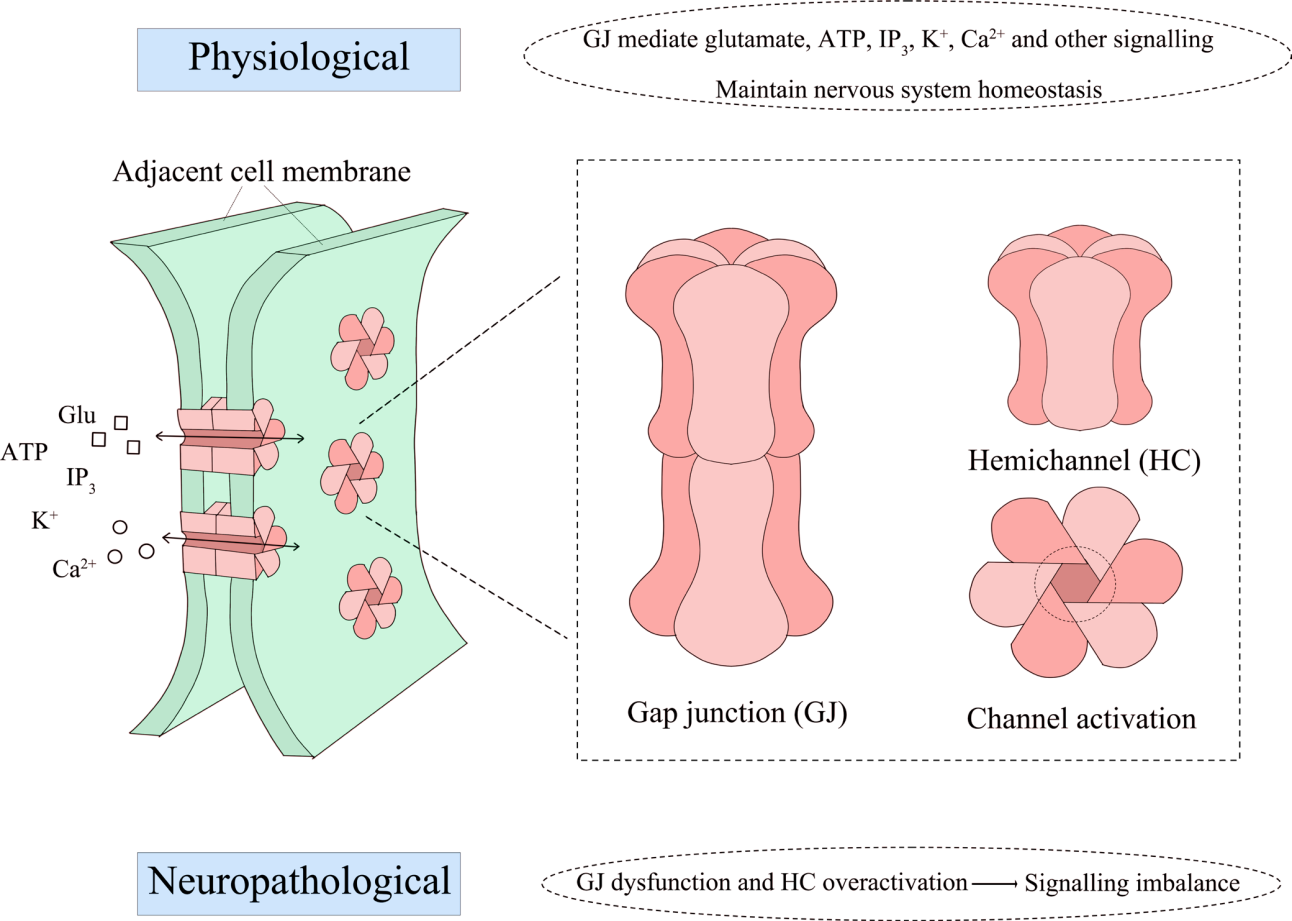
Schematic representation of astrocyte GJ structure. Astrocytes express a high density of GJs, consisting mainly of Cx43. GJs can connect adjacent cells and are the main structure for electrical transmission, metabolism, and ionic coupling between neighboring cells. Hexahedral structures on cells composed of Cx43 form Cx43 HC, and the HC of two neighboring cells dock to form GJs, which together mediate the exchange of information between cells. In pathological states, Cx43 GJ dysfunction and HC activation lead to impaired signaling, inducing a range of pathological injuries (neuroinflammation, Glu excitotoxicity, calcium overload, etc.).

Cx43 is a transmembrane protein that plays an important role in cell communication and physiological and pathological processes. Its main function is to form GJ between cells and mediate inter‐cell communication. It is the most direct and important way of signal conduction between cells, can quickly and reversibly realize the common response of adjacent cells to external signals [14]. Six Cx43 monomers form a connexon, also called HC, forming the central channel. The connexon of two adjacent cells dock to form a complete gap junction channel, whose opening and closing states are regulated by phosphorylation modifications, allowing direct communication between cells [6]. Cx43 not only participates in the exchange of substances between cells, but also participates in cell signaling and metabolic balance through HC functions. Under physiological conditions, Cx43 directly participates in action potential conduction in the electrical synaptic connections of neurons through GJ, shortening the excitation interval between neurons, thereby ensuring the stability of the environment outside the neuron and the stability of synaptic function [15]. In addition, Cx43‐mediated GJ allows astrocytes to functionally couple with other cells, such as oligodendrocytes and neurons, to form “glial syncytia” thereby maintaining the homeostasis of the nervous system [16]. Local calcium signals within the nervous system are able to propagate rapidly through Cx43‐mediated GJ in astrocyte networks, amplifying signals and coordinating large‐scale cellular responses [17]. Second messengers such as inositol triphosphate (IP_3_) can also spread through GJ, activating signaling pathways in distant cells [18]. Calcium ion signaling and metabolite exchange mediated by GJ can further regulate pre‐synaptic transmitter release and post‐synaptic receptor activation, affecting synaptic plasticity [19]. In the pathological state, Cx43 has a dual effect on the nervous system. Cx43‐mediated GJ participates in the diffusion of protective molecules to the extent of limiting the extent of damage [20]. However, Cx43‐mediated GJ dysfunction or aberrant expression is capable of triggering a greater degree of injury. For example, overactivated GJ and HC are capable of triggering calcium ion overload [21], glutamate (Glu) excitotoxicity [22], neuroinflammation [23], and other pathological damage. In various neurological diseases such as Alzheimer's, Parkinson's, and depression, abnormal expression of Cx43 and its mediated abnormalities in GJ and HC functions are often accompanied. In conclusion, Cx43 has a central role in the GJ assembly and function of astrocytes, and its regulation is not only crucial for the maintenance of neurological homeostasis, but also plays an important role in the onset and development of various neurological diseases. (Figure 1).

### 2.2 Intercellular Electrical and Chemical Signals Mediated by GJ in Astrocytes

Electrical and chemical signals between cells are the two main modes of intercellular communication, and they play a crucial role in multicellular organisms. Electrical signals include action potentials and local potentials, which directly transmit signals through changes in membrane potential caused by ion flow and rely on GJ, ion channels, and pumps on the cell membrane. In the nervous system, GJ that transmit electrical signals between cells are called electrical synapses and allow direct transmission of voltage signals between coupled cells [24]. Chemical signals transmit signals by releasing signaling molecules (such as neurotransmitters, hormones) to bind to target cell receptors. GJ provides a mechanism for inter‐cell communication between adjacent cells, allowing the direct exchange of messengers (such as calcium, nucleotides, IP_3_, and various metabolites) and electrical signals within cells, ultimately coordinating tissue balance, proliferation, differentiation, metabolism, cell survival, and death [25]. GJ channels formed between cells can coordinate electrical and metabolic activities between cells, while HC connect inside and outside cells and serve as diffusion paths for ions and small molecules [26].

Intercellular chemical signaling mediated by astrocytes through GJ is an important mechanism by which they coordinate metabolic activities, regulate neural network function, and respond to pathological stimuli. Chemical signaling relies on the direct diffusion of small molecules allowed by gap junction channels, such as metabolites, second messengers, and ions. Astrocyte‐mediated GJ can synergistically clear neurotransmitters such as Glu, prevent excitotoxicity, and maintain metabolic homeostasis. In addition, GJ regulates *N*‐methyl‐D‐aspartate receptor (NMDAR) activity and synaptic strength by coordinating D‐serine, ATP, IP_3_, etc., thereby regulating synaptic plasticity [27]. However, under pathological conditions, the function of GJ is impaired, which further exacerbates the damage to the nervous system. For example, under sustained stress, Cx43‐mediated HC activation on astrocytes leads to Glu accumulation in intercellular spaces, triggering excitotoxicity [22]. In addition, related studies have confirmed that astrocyte GJ dysfunction can aggravate neuroinflammatory responses [28, 29]. It is worth noting that the signals transmitted by astrocytes through GJ are not limited to simple chemical exchanges, but also include complex metabolism and electrical signal coupling.

**FIGURE 2:**
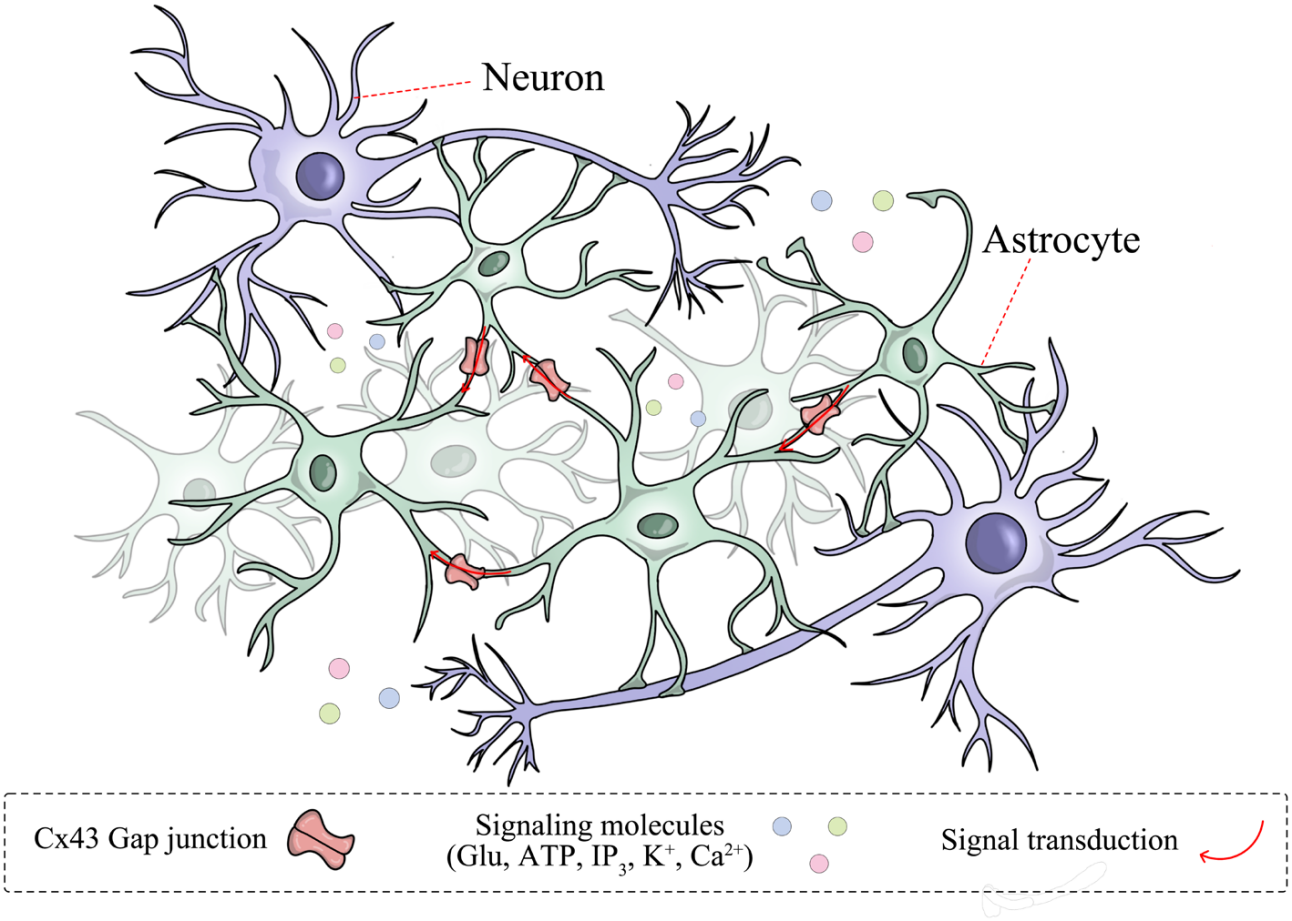
Schematic diagram of astrocyte network mediating electrical and chemical signaling. Astrocytes form a cellular network through Cx43‐mediated GJs, which ensure normal electrical activity in the nervous system by mediating K^+^ and Ca^2+^ signal propagation. In addition, astrocyte GJ synergistically conducts intercellular Glu, ATP, IP_3_, and other chemicals to maintain the homeostasis of the neural microenvironment and promote the establishment of synaptic function.

Astrocyte GJ mediating intercellular electrical signaling has been an important discovery in neuroscience research in recent years, and its mechanism involves a variety of molecular and physiological processes. Electrical signals rely on action potentials generated by ionic flow, which are rapidly conducted through neuronal axons and trigger chemical signal release at the presynapse, and are the “high‐speed channel” for information transmission in the brain. In patients with depression, electrical signaling in the brain is significantly altered, leading to structural disturbances in the brain network. Central to electrical signaling is the formation of low‐resistance channels by GJ, which allow the rapid flow of ions and currents between neighboring cells, thereby enabling electrical coupling of the astrocyte network. Astrocyte intercellular GJ channels allow K^+^ and Ca^2+^ to flow between cells, further maintaining homeostasis in the neural microenvironment [19]. Neuronal excitation triggers an increase in extracellular K^+^ concentration, and astrocytes can disperse excess K^+^ to the surrounding area through GJ, preventing the accumulation of local potassium ions from triggering neuronal overexcitation and ensuring normal neuronal electrical activity [30]. Astrocyte GJs are well able to reduce the depolarization of the cell membrane potential caused by the local elevation of extracellular K^+^ levels, which in turn efficiently maintains the K^+^ uptake drive and achieves isoelectricity in the cellular network, and thus can robustly maintain a constant extracellular environment for the key functions of neural circuits [31]. Furthermore, related studies have confirmed that astrocyte GJs play a crucial role in mediating intracellular Ca^2+^ signaling. Ca^2+^ acts as a second messenger, completes transport driven by intra‐ and extracellular ion concentration gradients, and participates in the generation and regulation of electrical signals through voltage‐gated channels, receptor‐gated channels, and other pathways. Astrocyte GJ allows intracellular Ca^2+^ signaling to neighboring resting cells, ultimately leading to the formation of intercellular Ca^2+^ waves [32]. Calcium waves are a mode of signal propagation that relies on GJ; when an astrocyte is stimulated, its internal Ca^2+^ concentration rises, transmitting the signal to neighboring cells via GJ, which triggers a series of calcium waves [33]. This calcium wave propagation is not only limited to localized regions but also spreads over long distances to the whole brain area and is able to propagate electrical signals in the form of waves through the astrocyte network [32]. During the development of depression, electrical signaling abnormalities are manifested as dysregulated neuronal excitability, disturbed neural oscillations, and impaired synaptic plasticity, and these alterations are brain‐region specific and frequency‐dependent. In conclusion, astrocytes are able to participate in the maintenance of intercellular ion homeostasis through gap junctions, modulate electrical signaling in the nervous system, and maintain neurohomeostasis to enhance the functional coordination of neural network. (Figure 2).

In summary, astrocytes play a key role in maintaining the homeostasis of the nervous system, coordinating neural network activities, and participating in pathological processes through intercellular electrical and chemical signaling mediated by GJs. Under physiological conditions, metabolic synergy and neural network function are maintained, whereas in disease, neural damage is exacerbated by GJ dysfunction that triggers some signaling abnormalities.

## 3 Role of Cx43 and Astrocyte GJ in Depression

### 3.1 Association of Abnormal Cx43 Expression and Function With Depression

The neuropathological effects of depression are associated with a variety of influencing factors, including neurotransmitters, inflammatory factors, and neurotrophic factors, but the specific neurobiological processes of depression have not been elucidated. In recent years, there has been increasing evidence that astrocyte dysfunction is strongly associated with the development of depression. Animal studies, postmortem brain analyses, and imaging studies in depressed patients have shown that astrocyte dysfunction is involved in the pathophysiology of MDD [10, 34]. The morphology and function of astrocytes were significantly altered in depressed patients compared to healthy controls [35]. Astrocyte morphological atrophy is considered to be the main histopathological feature of major depression in humans and animal models of depression [36]. Studies have shown that astrocyte density and their marker expression are significantly reduced in MDD [37]. In addition, considerable studies have confirmed that Cx43, which is synthesized in large quantities in astrocytes, and its mediators GJ and HC, which mediate energy exchange and effectively maintain physiological homeostasis, are involved in influencing the development of depression. For example, Sun et al. demonstrated that astrocyte GJ dysfunction plays an important role in the pathogenesis of depression [38]. Cx43 dysfunction can lead to depressive‐like behaviors by affecting the levels of inflammatory factors, neurotransmitters, and neurotrophins [14]. Furthermore, several autopsy studies have shown reduced expression of Cx43 in the locus coeruleus, frontal cortex, mid‐dorsal nucleus of the thalamus, and caudate nucleus in depressed individuals compared to healthy individuals [39, 40]. Expression of Cx30 and Cx43 was significantly reduced in the prefrontal cortex and hippocampus of mice with chronic social defeat stress depression model, and overexpression of Cx30 and Cx43 increased neuronal activity and suppressed depressive‐like behaviors [41]. Recently, studies have also demonstrated that specific knockdown of astrocyte Cx43 in the medial prefrontal cortex of mice induced depressive behaviors (including deprivation of pleasure and despair) and anxiety‐like behaviors [42]. In addition, a study using Cx43 transgenic mice demonstrated that conditional knockdown of Cx43 in astrocytes in the prefrontal cortex activated inflammatory signaling pathways, which in turn induced depressive behaviors in mice [43]. In summary, astrocyte dysfunction and abnormal Cx43 expression play key regulatory roles in the pathological mechanisms of depression.

### 3.2 Potential Mechanisms of Cx43 and Astrocyte GJ Dysfunction Leading to Depression

#### 3.2.1 Abnormal Transmission of Electrical Signals

Astrocytes are the most numerous glial cell type in the CNS and are primarily responsible for functions such as metabolic support, neuroprotection, neuronal survival, and synapse formation. They maintain the homeostasis of neural networks by communicating with neurons and other cells through structures such as GJ and HC [44]. Intercellular electrical signal transmission is influenced by various factors, including the generation and propagation of action potentials, ion channel regulation, and synaptic transmission. Research indicates that the generation and conduction of neuronal action potentials depend on the synergistic interaction of voltage‐gated ion channels [45]. The precise opening and closing of voltage‐gated ion channels control neuronal electrical signal conduction, thereby influencing neuronal excitability and neurotransmitter release [46]. The generation of action potentials is primarily influenced by the synergistic interaction of Na^+^ channels and K^+^ channels on the cell membrane. When a cell is stimulated, ion channels on the cell membrane open, allowing Na^+^ and K^+^ to flow across the membrane, causing rapid changes in membrane potential and forming an action potential [47]. When the action potential reaches the axon terminal, it triggers the release of neurotransmitters. These neurotransmitters diffuse across the synaptic cleft to receptors on the next neuron, triggering a new action potential. GJ allows direct electrical signaling between cells, whereas HC enables indirect communication through transmembrane transport of ions and molecules. Normal function of these structures is essential for maintaining the synchrony and stability of neural networks. Abnormal Cx43 expression and astrocyte GJ and HC dysfunction cause abnormal electrical signaling, which further leads to dysregulated neuronal‐astrocyte network activity.

K^+^ homeostasis in the brain is regulated by multiple activities of astrocytes, where locally increased K^+^ is able to be taken up by Kir channels and Na^+^/K^+^‐ATPase on astrocytes, with subsequent release of potassium ions into areas of low concentration via GJ‐mediated astrocyte network transfer [48]. There is a strong link between K^+^ signaling and Cx43‐mediated GJ, which is important for intercellular signaling in astrocytes in the nervous system [49]. Relevant studies have shown that in pathological states, astrocyte GJ dysfunction leads to impaired extracellular potassium ion clearance, depolarization of neuronal membrane potential, and malfunctioning of electrical signaling, resulting in neurological damage [30, 50]. Additionally, research has confirmed that upregulation of the inwardly rectifying potassium channel 4.1 (Kir4.1) channel expression on astrocytes promotes depressive phenotypes [51]. Inhibiting the gene expression of Kir4.1 in astrocytes can improve depressive phenotypes in mice [52]. Astrocyte GJs and K^+^ channels work together to maintain potassium ion homeostasis in the nervous system. K^+^ imbalance‐induced neural damage and abnormal electrical signal transmission may serve as potential pathological mechanisms underlying the onset and progression of depression.

Additionally, the close association of astrocytes with neuronal synapses and their syncytium‐like activity through GJ formation can facilitate their communication with distal brain regions via Ca^2+^ waves and maintain information transfer between different brain regions [53]. Calcium signaling is widely regarded as one of the most important messengers in the nervous system, playing a key role in regulating neuronal excitation and inhibition. Calcium signaling is essential for maintaining nervous system function, including the establishment of synaptic plasticity and signaling. Calcium signaling in astrocytes can occur independently of neuronal activity or after neurotransmitter release, including intrinsic Ca^2+^ oscillations within individual cells and Ca^2+^ waves that propagate from one type of astrocyte to another [32]. Related studies have confirmed that calcium wave conduction is significantly impaired with GJ blockers [54]. Furthermore, studies have shown that calcium wave conduction between astrocytes is significantly affected in the brains of Cx43 knockout mice [55]. In neurons and glial cells, abnormal Ca^2+^ signaling can lead to a variety of neuropathies, such as ischemia, depression, epilepsy, and autism [56]. Calcium overload in the nervous system can lead to various impairments of neural plasticity, protein synthesis, glial interactions, and other processes [57]. In addition, it can disrupt neural development, hinder mitochondrial function, and ultimately lead to cell death [58]. The nature of Ca^2+^ signaling in astrocytes is involved in acute and chronic changes in brain injury. When nerve tissue is damaged, astrocytes respond to the injury by changing the concentration of Ca^2+^ within them. Studies have shown that disruption of Ca^2+^ signaling in mouse astrocytes may lead to deficits in social interaction, depression‐like behavior, and abnormal synaptic structure and transmission [59]. In addition, related studies have shown that increased Ca^2+^ signaling is associated with astrocytes in response to neuronal overexcitation, amyloid plaques, ischemia, and tissue damage [60, 61, 62].

In addition to directly regulating Ca^2+^ signaling, astrocyte GJ can also indirectly affect calcium dynamics by regulating the release of chemical signals (Glu, ATP, cytokines) between cells. Related studies have shown that Glu, ATP, and many other inflammatory mediators in the extracellular environment can induce increased Ca^2+^ levels in glial cells [63]. Among the many receptors and channels that can mediate Ca^2+^ inward flow, Glu receptors mediate this process with greater specificity for neurons, and the NMDAR is uniquely hyperpermeable to Ca^2+^. Specialized astrocytes were found to be able to release gliotransmitters, such as Glu, through the glutamatergic receptor‐mediated calcium signaling pathway, thereby affecting neuronal excitability and synaptic transmission [4]. Glu released by astrocytes activates neuronal NMDAR, which significantly elevates intra‐neuronal Ca^2+^ concentrations due to its high permeability to Ca^2+^ [64]. In a state of depressive pathology, astrocyte GJ dysfunction and abnormal opening of HC channels occur, leading to impaired Glu clearance capacity. Additionally, activated half‐channels exacerbate Glu release, triggering Glu excitotoxicity. Excessive Glu release induces a strong influx of Na^+^ and Ca^2+^, disrupting ionic homeostasis and causing neuronal damage. Besides, it has been shown that γ‐aminobutyric acid (GABA) transporter proteins can be involved in mediating Ca^2+^ signaling in astrocytes through the uptake of GABA, which in turn affects neuronal function [65]. In astrocytes, GABA triggers Ca^2+^‐dependent Glu [66] and ATP [67] release, forming a feedback loop. ATP is an essential energy source for cells and can be released by neurons and astrocytes to mediate communication between astrocytes and neurons [68]. Studies have confirmed that ATP can be involved in the regulation of glutamatergic and GABAergic neurotransmission and plays a role in the onset and progression of depression [69]. As non‐electrically excitable cells, astrocytes initiate long‐distance Ca^2+^ waves via ATP release, activating neighboring cellular purinergic receptors and synchronizing transcellular signaling [21]. ATP plays a key role in calcium wave propagation in astrocytes and its effect on neural network function [70]. Studies have pointed out that ATP release mediated through GJ is one of the important mechanisms of calcium wave propagation [71]. The abnormal opening of HC mediates the release of intracellular ATP and Glu, which is an important danger signal. Under pathological conditions, Cx43 HC opens abnormally [72] and the resting membrane potential is disrupted, leading to abnormal release of Glu and ATP and activation of associated downstream damage [73]. For example, activation of pattern recognition receptors (such as purinergic receptors P2X7, P2Y, and Glu receptors) and inflammasomes on microglia and astrocytes initiates a neuroinflammatory cascade reaction. Furthermore, the occurrence of neuroinflammation can further enhance neurotoxicity and amplify abnormalities in astrocyte GJs and hemichannels, forming a positive feedback loop that continuously disrupts ion homeostasis and releases neuroactive substances. In conclusion, the rapid transmission of Ca^2+^ signaling through GJ between astrocytes affects neuronal function and may serve as a potential mechanism for the development of depression.

**FIGURE 3:**
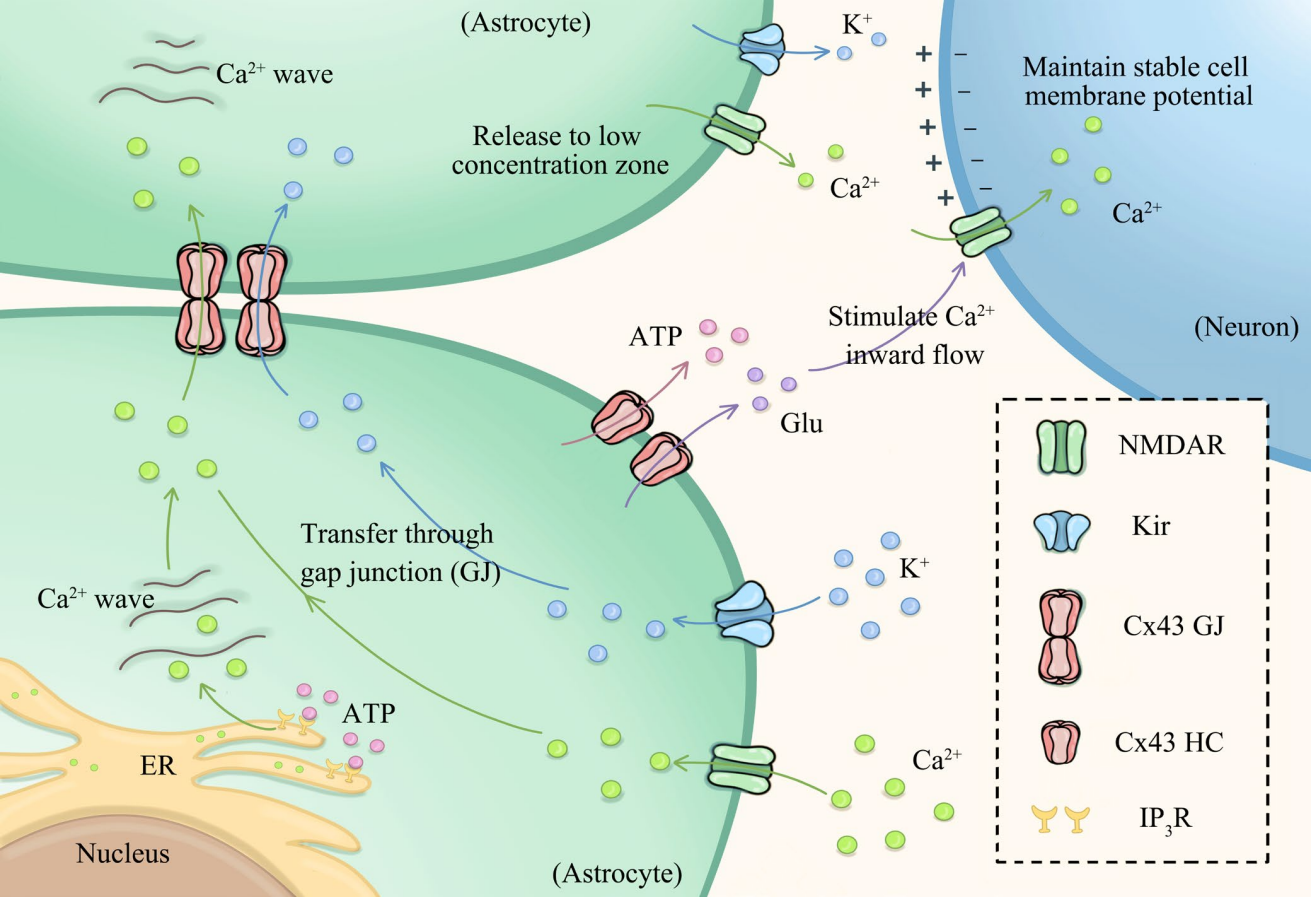
Schematic diagram of astrocyte GJ‐mediated K^+^ and Ca^2+^ conductance. Localized increases in K^+^ and Ca^2+^ in the interstitial space are taken up by ion channels on astrocytes and then diffuse through inter‐astrocytic GJ conductance to areas of low concentration to maintain neural homeostasis. In addition, cytosolic ATP and Glu can stimulate Ca^2+^ inward flow, triggering a local increase in intracellular calcium ions, and astrocyte GJ can prevent abnormal electrical signaling caused by calcium overload.

In summary, in neurons, the concentrations of K^+^ and Ca^2+^ jointly maintain the resting potential and the generation of action potentials. (Figure 3) The opening and closing of K^+^ channels determine changes in membrane potential, while Ca^2+^ plays a key role in neurotransmitter release. Together, they regulate neuronal excitability, synaptic transmission, and signal integration. Astrocyte Cx43 GJs participate in maintaining K^+^ and Ca^2+^ homeostasis, coordinating neural network activity, which may provide new insights into the pathological mechanisms of depression.

#### 3.2.2 Abnormal Chemical Signaling

The chemical signal transmission regulated by astrocyte GJ mainly includes the regulation of neurotransmitters, inflammatory factors, neurotrophic factors, ATP, and other small molecules. The imbalance of these substances is closely related to the occurrence and development of depression.

Relevant studies have confirmed that the abnormal expression of amino acid neurotransmitters is closely related to depression. Amino acid neurotransmitters play an important role in synaptic messaging, of which GABA is the main inhibitory neurotransmitter and Glu is the most abundant excitatory neurotransmitter. The two classes of neurotransmitters co‐ordinate with each other and work together to regulate the functioning of neurons and the cerebral cortex, thereby improving the mood of depressed patients [74]. GABA is produced by Glu through Glu decarboxylase and cooperates with Glu to maintain a dynamic excitation‐inhibition balance, which is considered to be the focus of the action of antidepressants [75, 76]. About 50% of Glu is involved in regulating synaptic transmission in the CNS and plays an important role in synaptic plasticity and neuron development [77]. There is a close link between excitatory neurotoxicity caused by excessive accumulation of Glu and the occurrence and development of depression. Under physiological conditions, Glu is absorbed by astrocytes and cleared from the synaptic gap by acting on the specific Glu transporter‐1 on astrocytes [78]. Astrocytes convert Glu to glutamine, diffuse it through GJ to neighboring cells, and ultimately return it to neurons for Glu biosynthesis. Under pathological conditions, excessive synthesis or release of Glu and abnormal expression of transporters lead to the accumulation of Glu in the synaptic cleft, leading to neuronal damage and cell death [79]. Dysfunction of GJ in astrocytes and activation of HC further lead to a decrease in Glu clearance in the synaptic space of cells, and the accumulation of Glu triggers neuroexcitotoxicity [22]. Disorders of GABAergic activity have been associated with many mental disorders, including anxiety‐related disorders and depression. Related studies have shown that depression is often accompanied by reduced GABA concentrations in the brain and plasma, as well as defects in the main receptor that mediates GABAergic activity. Cx43‐mediated GJ on astrocytes can be involved in affecting the transport of Glu and GABA, thereby preventing over‐activation of the nervous system [80]. In addition, GABA stabilizes and regulates the nervous system by reducing the excitability of the nervous system in the brain. The pathological accumulation of Glu can also lead to the obstruction of GABA synthesis. Changes in glutamatergic and GABAergic neurotransmission have often been observed in studies in patients with depression [81, 82]. Studies in stressed mice have shown that the development of depression‐like behavior is accompanied by GABA dysfunction in different brain regions [83]. In summary, Cx43‐mediated GJ dysfunction and HC activation on astrocytes can trigger an imbalance in the ratio of GABA to Glu, a decrease in GABA transport rate, and a surge in Glu, ultimately leading to neuronal damage and depression.

Neuroinflammation is a key factor in the pathogenesis of depression. It mainly contributes to the occurrence and development of depression by affecting the synthesis of neurotransmitters, activating the HPA axis, and affecting neurogenesis [84]. In addition, related studies have shown that the levels of inflammatory factors such as tumor necrosis factor‐alpha, interleukin‐1, and interleukin‐6 in the brain and plasma of patients with depression are elevated [85]. Many anti‐inflammatory drugs, such as celecoxib [86], aspirin [87] and tumor necrosis factor inhibitors [88], are used as supplements to treat depression. There is a close correlation between abnormal GJ function in astrocytes and increased release of inflammatory factors. This process involves pathological links such as dysregulated intercellular communication and amplification of immune responses. Under normal physiological conditions, astrocytes can transmit anti‐inflammatory signals and antioxidant molecules through GJ composed of Cx43, suppressing local inflammatory responses. On the contrary, excessive release of inflammatory factors can cause GJ dysfunction in astrocytes, down‐regulation of Cx43 gene expression, and anti‐inflammatory signals cannot be effectively transmitted through the GJ network, ultimately leading to damage to the nervous system [89]. In addition, pathological conditions cause abnormal opening of HC, which can directly release ATP, Glu, and inflammatory factors outside the cell, further amplifying the inflammatory cascade [29, 73]. Studies have shown that in the inflammatory process of various diseases, Cx43 HC is closely related to inflammatory responses and participates in the assembly and activation of the NLRP3 inflammatory body [23, 90, 91]. Related studies have confirmed that using Gap19 to block Cx43 HC opening can inhibit the inflammatory response and provide neuroprotective effects [92]. In vitro experimental results confirm that astrocytes Cx43, GJ, and HC play a key role in the neuroinflammatory response induced by oxygen–glucose deprivation/reperfusion injury [93]. Recently, studies have also confirmed that conditional knockout of Cx43 in astrocytes can induce anxiety‐ and depression‐like behaviors and lead to a decrease in ATP levels in prefrontal cortex cells. In addition, injection of exogenous ATPγS into the medial prefrontal cortex can alleviate depression‐ and anxiety‐like behaviors in mice with astrocyte‐specific Cx43 knockout [42]. In short, neuroinflammatory responses can affect the occurrence and development of depression in multiple ways, and abnormal function of astrocytes Cx43 GJ and HC plays a key role in this.

In summary, the GJ‐mediated electrical coupling network not only maintains the stability of the resting membrane potential of neurons, but also protects nervous system functions by regulating neurotransmitter precursors such as Glu and GABA and inflammatory factors. Electrical and chemical signal transmission mediated by astrocyte GJ‐mediated is very important for maintaining nervous system function. Abnormal signal transmission is closely related to the pathogenesis of depression and can be used as a potential mechanism for the occurrence and development of depression.

## 4 Depression Treatment Strategies

### 4.1 Targeting Cx43 and Astrocyte GJ Improves Depressive Symptoms

Cx43 is the core protein of GJ and HC in astrocytes and is involved in neurotransmitter homeostasis, inflammation regulation, and neuron protection. In depression treatment studies, related drugs improve depressive symptoms by improving the gap junction function of astrocytes. For example, initial studies have confirmed that the typical antidepressants duloxetine and fluoxetine can reverse Chronic Unpredictable Stress (CUS)‐induced astrocyte gap junction dysfunction in the prefrontal cortex [38]. Studies have shown that celecoxib, a drug that has shown antidepressant effects in clinical trials, can improve lipopolysaccharide—induced major depression, and its mechanism of action involves improving the gap junction function of astrocytes [94]. Higenamine can improve CUS‐induced depression‐like behavior in rats. It plays an antidepressant effect by improving the gap junction function of astrocytes, shortening the gap junction, increasing Cx43 expression, and reducing Cx43 phosphorylation [95]. Ginsenoside Rg1 can alleviate corticosterone (CORT)‐induced gap junction dysfunction of astrocytes and exert an antidepressant effect [96]. In addition, studies have shown that ginsenoside Rg1 improves CORT‐induced dysfunction of the astrocyte glutamatergic system by potentially reducing Cx43 phosphorylation and inhibiting the opening of HC, thereby improving GJC dysfunction [97]. Korean red ginseng can also improve the gap junction function of astrocytes, thereby alleviating depressive symptoms [98].

Similarly, quite a number of studies have shown that different drugs exert antidepressant effects through different mechanisms that affect the expression and function of Cx43. For example, Mahonia Alkaloids, the main active ingredients of the main drugs for treating depression in traditional formulations, were able to significantly increase the expression of Cx43 in the prefrontal cortex of the brain of depressed rats, which in turn modulated the Camp cyclic adenosine monophosphate Response Element Binding Protein/BDNF pathway to improve depressive behaviors [99]. Ginsenoside Rg1 reverses down‐regulation of astrocyte Cx43 biosynthesis in a depression model [100]. Studies have also confirmed that Rg1 can reduce depression by inhibiting Cx43 ubiquitization, thereby improving neuroinflammation [101]. Hypericin repairs gap junction dysfunction in rat models of CUS depression and reverses CORT‐stimulated phosphorylation of Cx43 in neonatal rat astrocytes, normalizing Cx43 expression [102]. Xiaoyao Powder can regulate the expression of Cx43 and improve the damage of hippocampal neurons in CUMS depression model rats [103]. Amitriptyline can improve the GJ channel between astrocytes by increasing the expression of Cx43, thereby improving depressive symptoms [104]. Antipsychotic clozapine, quetiapine, and brexpiprazole enhance Cx43 expression in astrocytes and thereby improve three‐sided synaptic glutamatergic transmission [105].

In summary, astrocyte Cx43, as a popular target for depression treatment, can significantly relieve depressive symptoms by improving astrocyte dysfunction and repairing Cx43‐mediated signal transmission. Further exploring its specific molecular mechanisms and how to more effectively regulate related pathways in the future will have important guiding significance for the development of new antidepressants.

### 4.2 Improve Depressive Symptoms by Targeting Electrical and Chemical Signals

Electrical and chemical signals are closely related in the nervous system. Electrical signals are transmitted through synapses, while chemical signals are mediated through neurotransmitters. As the main component of gap connections, Cx43 can promote the rapid transmission of electrical signals and affect the excitability of neurons by regulating chemical signals such as neurotransmitters. Therefore, regulating Cx43 function can not only improve the efficiency of electrical signal transmission, but also further relieve depressive symptoms through the regulation of chemical signals. Currently, there are many studies on interfering with electrical and chemical signal transmission to treat depression. In terms of chemical signals, intervention is mainly used to regulate the levels of neurotransmitters (serotonin, dopamine, Glu, etc.) in the brain to improve depressive symptoms. Currently, antidepressants often restore neurotransmitter balance by blocking transmitter reuptake, promoting release, or regulating receptor sensitivity. For example, selective 5‐hydroxytryptamine (5‐HT) reuptake inhibitors, antidepressants commonly used clinically in major depression, improve depressive symptoms by blocking neuronal reuptake of 5‐HT and increasing 5‐HT concentrations in the synaptic gap [106]. In addition, the novel fast‐acting antidepressant ketamine was able to improve the balance between Glu and GABA neurotransmission in MDD patients, which in turn repaired the associated network dysfunction [107]. Related traditional Chinese medicine also exerts antidepressant effects by regulating neurotransmitter levels. For example, Gastrodia elata water extract shows excellent antidepressant effect by regulating monoaminergic neurotransmission [108]. The classic traditional Chinese medicine prescription Danzhi Xiaoyao Powder and its active ingredients regulate the level of monoamine neurotransmitters, reduce neuroinflammation, and increase neurotrophic factors to produce neuroprotective antidepressant effects [109]. In addition, there has been an increasing number of drug studies that regulate Cx43 expression to influence chemical signaling and exert antidepressant effects. For example, ginsenoside Rg1 can improve neuroinflammation by inhibiting Cx43 ubiquitination and improve CORT‐induced astrocytic glutamatergic system dysfunction by reducing Cx43 phosphorylation levels and inhibiting HCs opening, thereby exerting antidepressant effects. Clozapine, quetiapine, and brexpilazepine participate in the antidepressant effects of drugs by influencing the activity of Cx43 HCs in astrocytes and Glu release. In summary, although the development of antidepressant drugs that directly target Cx43 still faces specific challenges, related drug research has demonstrated the potential of regulating the Cx43‐chemical signaling pathway.

In terms of electrical signals, the primary approach involves directly regulating brain region activity through physical means to improve neural circuit function, thereby alleviating depressive symptoms. Techniques such as repetitive transcranial magnetic stimulation (rTMS) and transcranial direct current stimulation (tDCS) have been shown to improve abnormal synchronization in neural networks by targeting and regulating the electrical activity of brain regions associated with depression, such as the prefrontal cortex and limbic system. For example, brain electrical stimulation techniques can regulate neural circuits associated with emotions in the brain, thereby improving depressive symptoms [110]. High‐frequency rTMS stimulation of the dorsolateral prefrontal cortex enhances functional connectivity between this region and the amygdala, correcting the overactivation of negative emotion processing pathways [111]. According to reports, tDCS is effective in treating suicidal tendencies in patients with bipolar disorder [112]. Additionally, studies have explored the antidepressant effects of regulating Ca^2+^ and K^+^ signaling. For example, D‐mannose promotes Ca^2+^ influx to enhance BDNF synthesis, thereby facilitating its rapid and sustained antidepressant effects [113]. Furthermore, curcumin has been shown to have antidepressant effects post‐stroke by preventing neuroinflammation caused by Ca^2+^ channel activation [114]. Puerarin can inhibit transient Ca^2+^ overload, reduce excitatory neurotransmitter levels and Ca^2+^ influx, and suppress apoptotic cascades, thereby exerting antidepressant effects [115]. Ginsenoside Rd. can exert antidepressant effects by inhibiting Ca^2+^ overload and mitochondrial dysfunction [116]. The clinical antidepressant fluoxetine acts on potassium channels to influence membrane potential. Ketamine and ritegabine act on voltage‐gated potassium channels regulated by chronic stress, enhancing the activity of Kv7 channels in ventral hippocampal glutamatergic neurons to produce sustained antidepressant effects [117]. Currently, research on drugs that directly target Cx43 to regulate electrical signals and exert antidepressant effects is still in its early stages. However, basic research has confirmed that Cx43 can regulate Ca^2+^ and K^+^ signal transmission and exert certain neuroprotective effects. This ion signal regulation‐mediated neuroprotective effect is closely related to the antidepressant mechanism. Therefore, clarifying the specific relationship between Cx43 and electrical signals is crucial for the development of novel antidepressant drugs.

To sum up, there is a complex interaction between electrical and chemical signals. GJ mediated by astrocytes Cx43 simultaneously regulates electrical and chemical signals, synergistically maintaining neural network homeostasis. Based on the target of Cx43GJ, we can deeply consider the changes in relevant electrical and chemical signals, further explore the interaction mechanism between electrical and chemical signals, and develop safer and more effective treatment options.

## 5 Conclusion

This review specifically explores the role of Cx43 and its regulated astrocyte GJ in the pathological mechanism of depression by regulating related electrical and chemical signals. Cx43 is the most abundantly expressed connexin in astrocytes, and its dysfunction is closely related to depression. Cx43‐mediated GJ and HC in astrocytes participate in regulating the transmission of electrical and chemical signals between astrocytes and neurons, and play a key role in maintaining the stability of nervous system functions. During the development of depression, astrocyte GJ dysfunction hinders some normal signal transmission, and abnormal synaptic transmission and neural network dysfunction cause a series of pathological damages, further exacerbating depressive symptoms. We explore the pathophysiological mechanisms of depression from the perspective of electrochemical signal transduction, representing a cutting‐edge interdisciplinary approach that integrates neurophysiology and neurochemistry. This approach transcends the limitations of the traditional “monoamine hypothesis,” focusing on the core processes of neuronal information transmission—the dynamic conversion and integration of electrical and chemical signals—to reveal deeper‐level neural circuit dysfunction underlying depression. Additionally, Cx43‐mediated electrical coupling and chemical coupling directly participate in the core regulation of neural homeostasis. Developing drugs that modulate its channel function or expression holds significant clinical application potential. Further exploring the interactions between Cx43 and its regulated astrocyte GJ channels with neural electrical and chemical signals could provide new insights for the diagnosis and treatment of depression.

## Author Contributions

Hongbin Wang critically reviewed the literature and wrote the manuscript. Naihong Chen, Songwei Yang, Yantao Yang, Meiyu Lin, Qidi Ai, Cong Chen, and Xuan Liu edited and revised important points. Yuting Lin, Zhifeng Tian, Zihan Yan, and Xuan Zeng contributed to investigation, methodology, and visualization. Naihong Chen, Songwei Yang, Yantao Yang, and Qidi Ai took responsibility for supervision and validation. Naihong Chen and Songwei Yang provided funding. All the authors read and approved the final manuscript.

## Conflicts of Interest

The authors declare no conflicts of interest.

## Data Availability

Data sharing not applicable to this article as no datasets were generated or analyzed during the current study.
